# Disaster Preparedness and Response Competencies Among Nurses in Disaster‐Affected Hospital: An Analysis of Knowledge, Attitudes, and Practices

**DOI:** 10.1155/nrp/5376735

**Published:** 2026-10-06

**Authors:** Cut Husna, Hilman Syarif, Irfanita Nurhidayah, Fikriyanti

**Affiliations:** ^1^ Department of Medical and Surgical Nursing Faculty of Nursing Universitas Syiah Kuala Banda Aceh 23111 Indonesia unsyiah.ac.id; ^2^ Department of Emergency Nursing Faculty of Nursing Universitas Syiah Kuala Banda Aceh 23111 Indonesia unsyiah.ac.id

**Keywords:** competencies, disaster, nurses, preparedness, response

## Abstract

**Introduction:**

The impact of disaster is significant on the physical, psychological, psychosocial, and spiritual conditions of survivors. Hospital requires adequate resources, including the readiness of nurses’ competencies in disaster management. This study aimed to compare nurses’ knowledge, attitudes, and practices (KAP) regarding disaster preparedness and response before and after a training intervention.

**Method:**

A quasi‐experimental study with one‐group pretest–posttest design was conducted. A total of 50 emergency and intensive care nurses were recruited using cluster random sampling. Data collection was performed using “Disaster Management Assessment Tool for Health Care Practitioners (DMAT‐HCP)” to identify KAP, willingness to continue practicing during disaster, and organization‐based disaster management. Data were analyzed using paired *t*‐test.

**Results:**

Significant differences were found between pre‐ and posttest results in nurses’ knowledge (*p* < 0.001), practices (*p* < 0.001), and willingness to continue practicing (*p* = 0.003). In contrast, no significant differences were observed in attitudes (*p* = 0.160) or organization‐based management (*p* = 0.112). Overall, the intervention significantly improved nurses’ competencies in disaster preparedness and response (*p* < 0.001; *d* = 0.60, 95% CI).

**Conclusions:**

The implementation of training interventions yielded significant enhancements in knowledge acquisition, practice, and willingness to continue performing duties during disaster scenarios. Notably, the study revealed that nurses’ attitudes and organization‐based management did not exert a statistically significant influence on disaster response outcomes. Conversely, targeted training and educational programs, including simulation‐based skills training, were found to be efficacious in augmenting nurses’ disaster competencies.

## 1. Introduction

The impact of disasters is significant on the physical, psychological, psychosocial, and spiritual conditions of survivors [1]. Hospital plays a crucial role in disaster management, and health workers, particularly nurses, are required to have adequate competencies in disaster preparedness and response [2]. Nurses, as the largest population and the frontline health workers during disaster, have an essential role and responsibility in disaster management [3].

Nurses’ competencies in disaster management refer to the Sendai framework for Disaster Risk Reduction 2015–2030 [4]. Moreover, nurses have a strategic role to collaborate with multidisciplinary teams to reduce the impact of disaster [5]. Several gaps were found in disaster preparedness, including the availability of early warning systems, public education and information, humanitarian aid procedures and health recovery [6], and lack of capability in epidemiological reporting and clinical decision‐making [7]. Disaster response competencies for handling vulnerable groups are still limited [8]. Therefore, the development of regulations, disaster management guidelines, and emergency and disaster training need to be prioritized for nurses [9]. The issue of nurses’ competencies in hospital remains debated 20 years after the 2004 Aceh earthquake and tsunami. Not all nurses have received specific emergency and disaster training. This situation leads to a lack of confidence in services, lack understanding of the duties, roles, and responsibilities, as well as limited knowledge, practices, and experience in real‐world disaster management.

During the preparedness, nurses engage in several activities, including identifying ethical practices, legal practices, accountability, communication, and information‐giving skills, as well as preparing plans for disaster management [7, 10]. Additionally, competencies are needed in vulnerability assessment, developing disaster prevention plans, organizing information systems, gathering resources, alarm systems, action mechanisms, community education and training, and disaster simulations.

The disaster response refers to the actual actions during disaster to rescue victims, meet the needs of victims, and reduce long‐term health impacts [11]. Nurses provide first aid, victim care, isolation of victims at risk of transmitting disease, aid distribution, psychological care, and special care for vulnerable populations [7, 12]. In the acute phase of response, competencies in disaster triage, treatment, and patient transfer are required [5]. Disaster triage, mental and psychological care, and life support are highly recommended [13]. For this reason, professional development efforts, mental health support, and collaborative efforts through training programs and policy development are essential [14]. During disasters and emergencies, nursing human resource management is often compromised, manifesting in several key issues. These include inadequate management of nursing personnel, ineffective command and control processes, inconsistent or absent presence of nursing staff, and insufficient motivational reinforcement mechanisms [15].

This study refers to the theoretical knowledge‐attitude‐behavior (KAB) framework that serves as a conceptual model for analyzing an individual’s psychological state. Because knowledge, attitude, and behavior form the core components of the learning process, they are essential considerations when designing educational interventions [16]. Sustainable knowledge positively and significantly influenced sustainable attitudes, subsequently driving sustainable behavior [17] and environmental concern strengthens the positive impact of system knowledge and effectiveness knowledge on individuals’ attitudes toward behavior [18]. According to the KAB framework, cognitive mastery of information (knowledge) serves as a prerequisite for shaping an individual’s internal evaluations and readiness (attitude), which ultimately manifests as observed actions (practices). In the context of healthcare and disaster response, knowledge regarding management protocols enhances practitioners’ perceived importance and confidence (attitude), thereby mitigating the “knowledge‐practice gap” and driving effective execution of clinical responses (practices).

Knowledge, attitudes, and practices (KAP) in disaster preparedness and response are vital competencies in disaster response. Structured disaster preparedness activities and training are highly recommended [19, 20]. Therefore, this study aimed to evaluate nurses’ KAP regarding disaster preparedness and response before and after a training program in hospital settings.

## 2. Materials and Methods

### 2.1. Study Design

A quasi‐experimental study with the one‐group pretest–post‐test design was conducted to compare nurses’ competencies in disaster preparedness and response through KAP. The design of study has potential threats to internal validity. We recognize that history and maturation pose potential threats to a single‐group pre–post design. To mitigate these risks, the interval between the pretest and post‐test was restricted to a brief 6‐week period, during which any external training‐related events were monitored and documented. Additionally, maturation effects were presumed to be negligible given the stable profile of the nurse participants. Furthermore, we assumed maturation effect was minimal because the participants were nurses with stable physical and psychological conditions.

### 2.2. Population, Sample, and Setting

The study was conducted from May to August 2025 in a provincial hospital of Aceh, Indonesia. The target populations were 184 nurses determined using cluster random sampling, with a total sample of 50 respondents. A medium effect size with a power of 0.8, a confidence interval (95 CI), and an alpha of 0.05 with a *d* value of 0.60 was used to determine the sample size [2]. The nurses were recruited in emergency department (ED) and intensive care (IC) because both units are the initial point to conduct intervention for emergency medical activities and clinical functions that are very vital in disaster response. The inclusion criteria of sample comprised: (1) ED or ICs nurses, (2) minimum work period of a year, (3) minimum diploma, and (4) not on leave/an assignment during the study period.

### 2.3. Measurement

This study was carried out using sociodemographic data of all respondents. Data were collected with “Disaster Management Assessment Tool for Healthcare Practitioners (DMAT‐HCP)” consisting of 48 items on 1–5‐point Likert scales [21]. The 12‐item knowledge subscale assesses healthcare professionals’ (HCPs’) comprehension of disaster management principles. Each item was scored on a 5‐point Likert scale, ranging from 1 (*no knowledge*) to 5 (*superior knowledge*). The 12‐item attitude subscale measures HCPs’ perceptions of key disaster management principles. Participants indicated their level of agreement using a 5‐point Likert scale (i.e., 1: strongly disagree, 2: disagree, 3: neutral, 4: agree, or 5: strongly agree). The 10‐item Practices subscale evaluates HCPs’ involvement in disaster management activities. Participants rated their level of engagement using a 5‐point Likert scale, ranging from 1 (*never*) to 5 (*very frequently*). The 6‐item willingness to continue practicing duties during disasters subscale evaluates HCPs’ readiness to perform their professional roles during crisis conditions. Responses were scored on a 5‐point Likert scale, ranging from 1 (*strongly unwilling*) to 5 (*strongly willing*). The 10‐item organization‐based disaster management subscale measures HCPs’ perceptions of institutional disaster preparedness. Each item was rated on a 5‐point Likert scale, ranging from 1 (*strongly disagree*) to 5 (*strongly agree*). The overall score for the DMAT‐HCP (48 items) ranges from a minimum of 48 to a maximum of 240 (mean range: 1.00–5.00), with higher scores indicating higher proficiency in disaster management.

The instrument of DMAT‐HCP passed structural validity, construct validity, and internal consistency testing. The internal consistency coefficients, measured by Cronbach’s alpha, varied from 0.89 for the attitude section to 0.97 for organization‐based management, yielding an overall value of 0.90 for the DMAT‐HCP. We clarify that the validity and Cronbach’s alpha reliability values reported in the manuscript were adopted from the original validation study by Elshami et al. (2025). To adapt the instrument for our study context, we followed a rigorous translation and adaptation process. First, the instrument underwent standard forward‐and‐back translation by independent translators to preserve semantic consistency. Second, it was evaluated by a panel of three experts from the Faculty of Nursing, Universitas Syiah Kuala, Banda Aceh, Indonesia, who possess extensive academic and clinical expertise in medical‐surgical and emergency nursing. During this review, the items were meticulously examined, evaluated, and refined to ensure linguistic clarity, cultural appropriateness, and contextual relevance for local hospital settings. The overall item‐level content validity index (I‐CVI) was 0.95.

### 2.4. Intervention and Data Collection

The intervention was conducted over six sessions of educational and training programs, with each being a week for one group. Each session consisted of 4 h provided material related to the KAP competencies of disaster preparedness and response. This includes session 1: Providing information and study objectives, and pretest; session 2: Ethical practices, accountability, ethical dilemmas in disaster and the role of nurses, communication and reporting in disaster and the chain of command; session 3: Triage and basic first aid in disaster; session 4: Patient care, isolation of victims in disaster, and psychological care in disaster; session 5: Patient transfer in disaster, and hospital disaster plan; and session 6: Concept and tabletop exercise simulation, and post‐test to assessing the outcomes. Subsequently, study information and objectives were provided, respondents signed written informed consent to participate voluntarily, and data confidentiality was guaranteed by providing anonymity and coding.

To maximize participant attendance during training, daily attendance was strictly monitored, and training schedules were adjusted according to the participants’ availability for each session. The training intervention consisted of six weekly sessions, with each session lasting 4 h and combining theoretical instruction with hands‐on practice delivered by field experts. To facilitate participation, schedules were coordinated with hospital administration to align with the nurses’ shift rotations. The overall session attendance rate was 98.3%. Three participants were dropped from the final analysis due to insufficient attendance, leaving a final sample of 50 nurses (*n* = 50) who fully completed the intervention and evaluations. To ensure trainer competence, this study involved a research team of four emergency nursing experts from the Faculty of Nursing at Universitas Syiah Kuala, Banda Aceh, Indonesia, all of whom possess a high level of professional qualifications. Implementation fidelity was ensured and monitored through a standardized protocol and post‐session checklists to evaluate training delivery consistency.

### 2.5. Data Analysis

After six follow‐up sessions of completion training programs involving 50 respondents in one pretest–post‐test intervention group, data analysis was conducted. Collected data were analyzed by a paired *t*‐test statistical method using SPSS version 20.0. The paired *t*‐test requires several key assumptions: paired or dependent observations, a continuous dependent variable, normally distributed differences between pairs, and the absence of significant outliers in those differences. Normality of the paired differences was evaluated using the Shapiro–Wilk test prior to conducting the paired *t*‐test, confirming that the distribution met the assumption of normality (*p* > 0.05). No missing data were present in the analysis; all enrolled participants completed both pre‐ and post‐assessments.

The flowchart of the study is shown in Scheme 1.

**Scheme 1:**
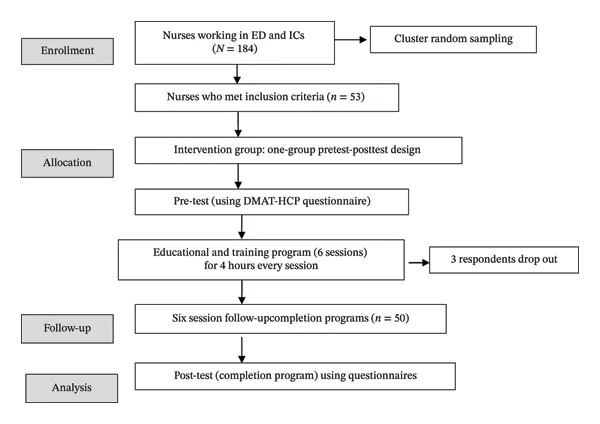
The flowchart of the study.

### 2.6. Ethical Consideration

This study was conducted in accordance with relevant ethical guidelines and received formal approval from the Ethics Committee of a Provincial Hospital (Ref. No. 230/ETIK‐RSUDZA/2025). Written informed consent was obtained from all participants prior to their inclusion in the study.

## 3. Results

The demographic data of respondents consisted of age, gender, last education, workspace (ward), length of work, experience in caring for disaster survivors, experience with disaster, attending emergency/disaster training, and type of emergency/disaster training. Detailed sociodemographic data of the nurses are presented in Table 1.

**TABLE 1:** Sociodemographic data of respondents (*n* = 50).

Characteristics	Frequency	Percentage
Age (year):		
Mean ± SD	33.8 ± 4.7	
Gender:		
Male	19	38
Female	31	62
Last education:		
Diploma	14	28
Bachelor	34	66
Master nursing	2	6
Length of work (year):		
Mean ± SD	6.7 ± 4.3	
Min	2	
Max	25	
Workspace:		
High care unit medical (HCU Med)	11	22
High care unit surgical (HCU Surg)	8	16
Intensive care unit (ICU)	19	38
Emergency department (ED)	12	24
Experience in caring for disaster survivors:		
Yes	26	52
No	24	48
Experience with disaster:		
COVID‐19	23	88.46
Flash floods	1	3.85
Earthquakes	1	3.85
Tsunami	1	3.85
Attended emergency/disaster training:		
Yes	11	22
No	39	78

The score pretest of competencies among nurses is shown in Table 2; knowledge shows a fairly wide range, with a moderate average and variability. The attitudes toward disaster management have a narrower range, with a high average and low variability.

**TABLE 2:** Pretest of mean and standard deviation of nurses’ competencies (*n* = 50).

Variable	Min	Max	Mean	Std. deviation
Knowledge	19	51	33.52	7.94
Attitudes	34	55	45.72	4.78
Practices	10	42	23.56	9.34
Willingness to continue practicing duties during disaster	9	30	21.20	5.40
Organization‐based disaster management	26	50	40.66	6.15

The practice in disaster preparedness and response have a fairly wide range, with a moderate average and variability. The post‐test mean and SD of nurses’ competencies are shown in Table 3.

**TABLE 3:** Posttest of mean and standard deviation of nurses’ competencies (*n* = 50).

Variable	Min	Max	Mean	Std. error	Std. deviation
Knowledge	21	55	46.22	1.30	9.24
Attitudes	15	55	47.68	1.13	8.05
Practices	12	50	33.28	1.62	11.50
Willingness to continue practicing duties during disaster	10	30	24.30	0.74	5.23
Organization‐based disaster management	10	50	42.98	1.05	7.49
Nurses’ competencies in disaster preparedness and response	105	240	194.46	4.10	29.04

Table 4 presents a significant difference between the actual and expected values for several variables, such as knowledge, practice, and willingness to perform duties during a disaster. This shows a significant change observed between pre‐ and post‐test of the intervention on improving nurses’ competency scores.

**TABLE 4:** The differences between pretest and posttest results of nurses’ competencies in disaster preparedness and response (*n* = 50).

Variable	Mean	Std. deviation	Std. error mean	95% confidence interval		*t*	*p*
				Lower	Upper		
Knowledge	−12.70	12.26	1.733	−16.18	−9.21	−7.324	< 0.001
Attitudes	−1.96	9.72	1.374	−4.72	0.80	−1.425	0.160
Practices	−9.72	14.64	2.071	−13.88	−5.55	−4.692	< 0.001
Willingness to continue practicing duties during disaster	−3.10	6.88	0.973	−5.05	−1.14	−3.184	0.003
Organization‐based disaster management	−2.32	10.14	1.434	−5.20	0.56	−1.617	0.112
Nurses’ competencies in disaster preparedness and response	−29.80	35.71	5.05	−39.94	−19.65	−5.901	< 0.001

The KAP and nurses’ competencies in disaster response are depicted in Figure 1.

**FIGURE 1:**
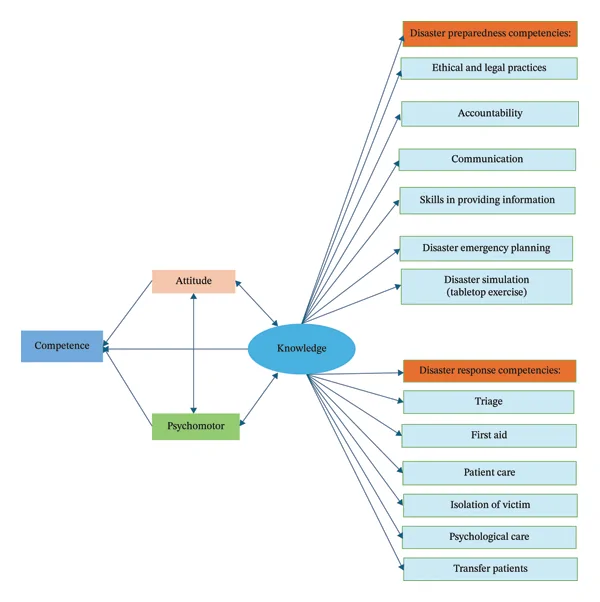
Nurses’ disaster preparedness and response competencies (KAP).

## 4. Discussion

Over the past few decades, disasters have impacted on physical, psychological, social, and spiritual conditions, placing HCPs, particularly nurses, as frontline health workers in disaster situations, ensuring an adequate response.

Nurses’ competencies in disaster preparedness and response include KAP. The knowledge of nurses is crucial, particularly in understanding disaster management concepts, including reporting procedures, disaster awareness campaigns, collaboration in mitigation, role clarity, access to information, organizational protocols, and post‐disaster response systems and roles [21].

In this study, nurses’ competencies regarding disaster preparedness consisted of ethical and legal practices, accountability, communication, skills in providing information, disaster management planning, and disaster simulation. Meanwhile, competencies in disaster response comprise triage, first aid, patient care, isolation of victims, and psychological care. The results showed that nurses’ knowledge had significant difference regarding disaster preparedness and response in hospital‐based training. These support the perception that nurses sufficiently equipped with continuous emergency and disaster training could have improved specific competencies in serving vulnerable groups and understanding emergency information in disaster management [22], overcome various challenges, and increased resilience to disaster [23]. Nurses’ competencies should be improved through regular and structured formal preparedness training [24].

Core competencies for nurses are essential in facing future disaster [25]. There is a need to improve nurses’ knowledge through disaster preparedness educational and training programs, which could be arranged in hospital policies and programs [26]. Additionally, perceived competencies were influenced by education, disaster simulation experience, and plan awareness. Nurses’ competencies were related to educational qualifications, awareness of management plans, and disaster simulation experience [27].

Nurses’ attitudes refer to confidence in implementing management procedures and confidence in carrying out tasks independently during a disaster. The results show no difference between the pretest and post‐test scores of attitudes toward preparedness and response. These relate to the fact that although nurses’ knowledge and practices increase because of training, there is no improvement in the nurses’ attitude. Nurses’ beliefs showing similar perceptions before and after the training could be associated with the results. The majority of nurses had experience in caring for disaster survivors. Consequently, nurses have adequate attitudes and beliefs regarding disaster preparedness, and response from last experienced before training was collected. Attitudes greatly determine the adequacy of self‐efficacy in disaster [28].

The results show that disaster training has a significant improvement following the intervention on nurses’ practices. The observed improvement in practice scores may be related to interactive learning methods, such as tabletop exercises, role‐playing, and case studies, which offer participants practical, real‐world experiences. A previous study reporting disaster training had a positive association with nurses’ clinical competencies, decision‐making skills, and rapid response in emergency situations [29, 30] Therefore, regular disaster‐based training and simulations are crucial for evaluating and strengthening the roles and functions of nurses to response [31]. The training had a broad impact on mastery of disaster management skills [32, 33]. The skills are core competencies of disaster nursing according to the International Council of Nurses (ICN) [34].

The result corresponds with previous studies emphasizing that enhancing competencies through disaster education and training related to increased willingness to respond [35, 36]. Additionally, nurses with real‐life disaster response experience showed a higher level of willingness to perform tasks compared to lacking the experience. Direct exposure, simulation, and experiential training may enhance nurses’ self‐confidence and psychological preparedness [37]. Globally, willingness to respond remains a challenge, specifically when nurses feel underequipped organizational support. Comprehensive and in‐house training is crucial for strengthening readiness and willingness [29]. Preparedness training plays a crucial role in improving practical skills as well as fostering nurses’ commitment and willingness to continue duties during disaster [5, 34]. Also, a standardized, foundational knowledge framework enhances disaster response processes. The implementation and evaluation of discussion‐based and operation‐based exercises significantly augmented knowledge development and fostered behavioral change among prehospital nurses, thereby facilitating a standardized emergency response [38].

The effectiveness of hospital disaster management is more determined by institutional policies, management plans, and cross‐professional coordination than by individual training [19]. The greatest challenges in disaster preparedness are limited organizational support, coordination, logistics, and management policies [29]. Therefore, the obtained results are consistent with the previous study reporting that organizational‐based preparedness is influenced by hospital systems and policies than by improving personal competencies.

Nurses’ competencies could be improved through training and require structural interventions, including hospital management. These results correspond with ICN recommendations emphasizing that organizational‐based preparedness can only be improved when there is integration between individual training and organizational policies [33]. Limited managerial support and the absence of large‐scale simulations are often major obstacles to the effectiveness of disaster management [29]. Additionally, disaster management preparedness was reported to be ineffective, related to lack of cultural awareness, delays in response to incidents, and poor coordination with local governments and international agencies [34].

This confirms that improvement of competencies is not only determined by knowledge but also by practical skills. Other studies emphasize practical skills such as triage, first aid, patient isolation, and psychological care that play important roles in a successful disaster response performance [13, 14]. Disaster‐related skills were most significantly associated with nurses’ perceptions of preparedness [39]. Disaster‐related experience most strongly influences the main competencies of nurses, followed by knowledge [40, 41]. Knowledge and practical skills to disaster scenarios, participation in rescue and emergency drills, and prior training were key factors influencing competencies among hospital nurses [42]. The ICN mentioned that strengthening nurses’ capacity in disaster preparedness should comprise KAP in a balanced manner to improve competencies [20].

The results confirm positive nurses’ attitudes toward disaster management: the higher the competencies in carrying out roles in emergency situations. These correspond with a study reporting that attitudes are an important predictor of competence and can be improved through training interventions. Therefore, positive attitudes will promote increased nurses’ competencies [43]. Disaster preparedness attitudes contribute significantly to the nurses’ core competencies [41]. Positive attitudes are capable of fostering the development of competencies [44]. Positive attitudes are important for mental readiness of nurses, which contributes to increasing practical competencies.

## 5. Conclusion

Disaster management requires adequate preparedness and response from all healthcare providers, including nurses as frontline healthcare workers. Nurses’ education and training program intervention successfully improved nurses’ knowledge, practices, and willingness to perform duties in disaster situations. However, the variables of attitudes and organization‐based disaster management did not show significant differences as measured by the pretest and post‐test due to the association with several factors such as perceived about nurses’ competencies and perceived roles in preparedness and response to disaster. Well‐preparedness related to organization‐based disaster management and willingness to continue practicing duties during disaster to be policy implications will be emphasized in the future.

### 5.1. Limitation of the Study

This study has several limitations. First, the use of a quasi‐experimental one‐group pretest and post‐test design without a control group limits the ability to establish a definitive causal relationship between the disaster preparedness training and improvements in nurses’ KAP. Therefore, potential threats to internal validity, such as history, maturation, and testing effects, cannot be fully excluded. Second, the study was conducted at a single hospital in a disaster‐affected area with a relatively small sample size, which may limit the generalizability of the findings to broader populations or different institutional settings. Third, the absence of long‐term follow‐up precludes evaluating the sustained associations on the intervention over time. Additionally, the reliance on self‐reported data introduces the potential for response or social desirability bias. Finally, repeated administration of the assessment tools may have introduced testing or recall effects, potentially influencing postintervention outcomes. Despite these limitations, this study provides preliminary evidence on the associations of disaster preparedness training on nursing competencies. Future studies with a randomized controlled trial (RCT) design and multicenter settings are recommended to confirm these findings.

## Author Contributions

Cut Husna, Irfanita Nurhidayah, and Hilman Syarif wrote the protocol and monitored the data collection. Irfanita Nurhidayah and Fikriyanti collected the data and assisted with statistical analysis. Cut Husna and Irfanita Nurhidayah conducted the primary data analysis and drafted the initial manuscript. Cut Husna, Hilman Syarif, and Fikriyanti contributed to the critical review of the manuscript.

## Funding

This study was funded with an award granted by the Directorate of Research and Community Services, Ministry of Higher Education, Science and Technology, Indonesia, number: 113/C3/DT.05.00/PL/2025, and the Institute for Research and Community Services of Universitas Syiah Kuala, Banda Aceh, Indonesia, number: 82/UN11.L1/PG.01.03/DPPM/2025.

## Disclosure

All authors have approved the final version of this article.

## Conflicts of Interest

The authors declare no conflicts of interest.

## Data Availability

The data that support the findings of this study are available from the corresponding author upon reasonable request.

## References

[1] Husna C. , Kamil H. , Yahya M. et al., Exploring Perspectives of Disaster Survivors on Islamic-Based Nurses’ Disaster Response Competencies: A Study From Disaster Front Area, Journal of the Liaquat University of Medical and Health Sciences. (2024) 23, 125–133, 10.22442/jlumhs.2024.01080.

[2] Husna C. , Kamil H. , Yahya M. , and Tahlil T. , An Intervention Program to Improve Nurses’ Competencies in Disaster Response: A Mixed-Methods Study Protocol, Belitung Nursing Journal. (2020) 6, no. 3, 85–90, 10.33546/bnj.1081.

[3] International Council of Nurses , 2022, International Council of Nurses Core Competencies in Disaster Nursing: Competencies for Nurses Involved in Emergency Medical Teams (Level III).

[4] United Nations International Strategy for Disaster Reduction , Sendai Framework for Disaster Risk Reduction 2015-2030, 2015, 1st edition, United Nations.

[5] World Health Organization and International Council of Nurses , ICN Framework of Disaster Nursing Competencies, 2009, 1st edition, World Health Organization and Western Pacific Region.

[6] Pelupessy W. V. and Silverman R. M. , Exploring Indonesian Coastal Communities’ Responses to the 2019 Ambon Earthquake and Preparedness for Future Disasters, International Journal of Disaster Risk Reduction. (2024) 114, 10.1016/j.ijdrr.2024.104961.

[7] Husna C. , Miranda F. , Darmawati D. , and Fithria F. , Emergency Preparedness Information Among Nurses in Response to Disasters, Enferm Clin. (2022) 32, S44–S49, 10.1016/j.enfcli.2022.03.016.

[8] Husna C. and Azhari A. P. , Analisis Kompetensi Respon Bencana Pada Perawat Di Puskesmas Kabupaten Bireuen, Aceh (Disaster Response Competency Analysis of Nurses at the Bireuen District Health Center, Aceh), Jurnal Ilmu Keperawatan Medikal Bedah. (2021) 4, 69–83, 10.32584/jikmb.v4i1.893.

[9] Husna C. , Yahya M. , Kamil H. , and Tahlil T. , The Impact of Islamic-Based Disaster Response Competencies Program on Nurses: A Computer-Based Training Randomized Controlled Trial, The Open Nursing Journal. (2021) 15, no. 1, 433–443, 10.2174/1874434602115010433.

[10] Husna C. , Firdaus R. , Wardani E. , and Jannah S. R. , Disaster Preparedness Among Disaster Management Agency Officers: A Study From Rural and Urban Areas in Aceh, Indonesia, International Journal of Disaster Resilience in the Built Environment. (2022) 13, no. 4, 484–497, 10.1108/IJDRBE-02-2021-0015.

[11] International Council of Nurses , 2019, Core Competencies in Disaster Nursing Version 2.0. Switzerland.

[12] Ascolese M. A. , Keyes K. A. , Ropero-Miller J. D. , Wire S. E. , and Smiley-McDonald H. M. , Mass Fatality and Disaster Response Preparedness Across Medical Examiner and Coroner Offices in the United States, Forensic Science International. (2024) 8, 10.1016/j.fsisyn.2024.100462. PMC10909695 38439787

[13] Hasan K. , Hoque M. , Mahatasim M. et al., Factors Influencing Disaster Response Self-Efficacy Among Registered Nurses in Bangladesh, Progress in Disaster Science. (2024) 23, 10.1016/j.pdisas.2024.100341.

[14] Ward A. , Martin S. , Psych G. et al., Enhancing Primary Healthcare Nurses’ Preparedness for Climate-Induced Extreme Weather Events, Nursing Outlook. (2024) 72, no. 5, 10.1016/j.outlook.2024.102235. 39004069

[15] Moghadam M. N. , Kamali H. , Amiresmaili M. , and Moradi S. M. , Perspectives and Experts of Human Resource Management in Nursing During Disasters and Emergencies: A Qualitative Content Analysis, Nursing Research and Practice. (2025) 2025, no. 1, 10.1155/nrp/9921673. PMC12520809 41098316

[16] Schrader P. G. and Lawless K. A. , The Knowledge, Attitudes, & Behaviors Approach: How to Evaluate Performance and Learning in Complex Environments, Performance Improvement. (2004) 43.

[17] Lučić A. and Uzelac M. , Sustainable Household Capability of Young Adults: Role of Parental Norms, Heliyon. (2024) 10, no. 7, 10.1016/j.heliyon.2024.e29219. PMC11015442 38617972

[18] Gong Q. , Liu Y. , Liu X. , Wang F. , and Zhu H. , Influence of Multi-Dimensional Environmental Knowledge on Residents’ Waste Sorting Intention: Moderating Effect of Environmental Concern, Frontiers in Psychology. (2022) 13, 10.3389/fpsyg.2022.957683. PMC9745202 36524171

[19] Richmond J. G. , Tochkin J. , and Hertelendy A. J. , Canadian Health Emergency Management Professionals’ Perspectives on the Prevalence and Effectiveness of Disaster Preparedness Activities in Response to COVID-19, International Journal of Disaster Risk Reduction. (2021) 60, 10.1016/j.ijdrr.2021.102325. PMC9764162 36570631

[20] Khandan M. , Ghorbani Z. , Golestani M. , and Moradi F. , The Competence of Nurses Caring for COVID-19 Patients Regarding Disaster Management: Structural Equation Modeling of Knowledge, Attitude, and Performance, Heliyon. (2024) 10, no. 15, 10.1016/j.heliyon.2024.e35568. PMC11336690 39170153

[21] Elshami S. , Ibrahim M. I. M. , Abdel-Rahman M. E. , Rahim H. A. , and Mukhalalati B. , Developing and Evaluating a Disaster Management Assessment Tool for Health Care Practitioners, BMC Emergency Medicine. (2025) 25, no. 1, 10.1186/s12873-025-01199-8. PMC11887106 40050754

[22] Asman A. , Mansoben N. , Amir H. et al., Nurse Preparedness in CHCs: Evaluating Disaster Response Competencies, Universal Journal of Public Health. (2024) 12, no. 3, 515–521, 10.13189/ujph.2024.120309.

[23] Akkaya G. , Akkuş Y. , Parlak A. G. , and Karacan Y. , Determination of the Relationship Between Competencies in Disaster Nursing Management and Perceived Stress: the Intermediary Role of Resilience, International Emergency Nursing. (2025) 78, 1–6, 10.1016/j.ienj.2024.101530. 39591683

[24] Chegini Z. , Arab-Zozani M. , Kakemam E. , Lotfi M. , Nobakht A. , and Aziz Karkan H. , Disaster Preparedness and Core Competencies Among Emergency Nurses: A Cross-Sectional Study, Nursing Open. (2022) 9, no. 2, 1294–1302, 10.1002/nop2.1172. PMC8859035 34985209

[25] Jaafar S. A. , Al-Jubouri M. B. , Mousa A. M. et al., Disaster Preparedness and Core Competencies in Iraqi Nurses, Iranian Journal of War and Public Health. (2024) 16, 355–361, 10.58209/ijwph.16.4.355.

[26] Şermet K. Ş. and Erdoğan E. G. , Disaster Management Competence, Disaster Preparedness Belief, and Disaster Preparedness Relationship: Nurses After the 2023 Turkey Earthquake, International Nursing Review. (2025) 72, no. 1, 10.1111/inr.13020. PMC11840655 38973366

[27] Winarti W. and Gracia N. , Exploring Nurses’ Perceptions of Disaster Preparedness Competencies, Nurse Media Journal of Nursing. (2023) 13, no. 2, 236–245, 10.14710/nmjn.v13i2.51936.

[28] Sanjaya I. W. , Permana I. , and La E. A. R. , The Effect of Self Efficacy and Attitudes on Nurses’ Preparedness, Indonesian Journal of Global Health Research. (2025) 7, 263–270, 10.37287/ijghr.v2i4.250.

[29] Labrague L. J. and Hammad K. , Disaster Preparedness Among Nurses in Disaster-Prone Countries: A Systematic Review, Australasian Emergency Care. (2024) 27, no. 2, 88–96, 10.1016/j.auec.2023.09.002. 37778913

[30] AlOtaibi S. S. , Almutairi H. A. , Alotaibi M. K. , Alharbi K. , and Bahari G. , Enhancing Nurses’ Disaster Management and Preparedness: Evaluating the Effectiveness of an Online Educational Program Through a Quasi-Experimental Study, Risk Management and Healthcare Policy. (2024) 17, 101–111, 10.2147/RMHP.S446704. PMC10788064 38222797

[31] Alim S. , Kawabata M. , and Nakazawa M. , Evaluation of Disaster Preparedness Training and Disaster Drill for Nursing Students, Nurse Education Today. (2015) 35, no. 1, 25–31, 10.1016/j.nedt.2014.04.016. 24832532

[32] Xia R. , Li S. , Chen B. , Jin Q. , and Zhang Z. , Evaluating the Effectiveness of a Disaster Preparedness Nursing Education Program in Chengdu, China, Public Health Nursing. (2020) 37, no. 2, 287–294, 10.1111/phn.12685. 31709640

[33] International Council of Nurses , Core Competencies in Disaster Nursing Version 2.0, 2019, 1st edition.

[34] Stewart D. , Gebbie K. , and Hutton A. , 2022, Core Competencies in Disaster Nursing.

[35] Feng J. , Zhang C. , Fang S. , Zhao R. , Wang H. , and Li D. , Influencing Factors of Nurses’ Disaster Preparedness: A Systematic Review and Meta-Analysis, BMC Public Health. (2025) 25, no. 1, 10.1186/s12889-025-23981-w. PMC12326711 40770316

[36] Motsepe T. L. and Schmollgruber S. , Emergency Nurses’ Disaster Preparedness Competencies: A Focused Mapping Review and Synthesis, Health SA. (2025) 30, no. 0, 10.4102/hsag.v30i0.2770. PMC11830908 39968244

[37] Ulupınar F. , Erden Y. , Duraklı M. , and Şahiner Y. A. , Disaster Nursing Preparedness and Response Competency: A Comparative Study of Nurses Instantly Deployed and Not Deployed in the 2023 Kahramanmaraş Earthquakes in Turkey, Disaster Medicine and Public Health Preparedness. (2025) 19, 10.1017/dmp.2025.10079. 40524250

[38] Aminafshar A. , Khosravizad A. , Sahebi A. et al., Identifying Key Indicators and Components of Earthquake Preparedness Exercises in the Prehospital Setting: An Exploratory Content Analysis From Paramedic Nurses’ Perspectives, Nursing Research and Practice. (2026) 2026, no. 1, 10.1155/nrp/1785701. PMC12853389 41625018

[39] Khairina I. , Nelwati N. , Maisa E. A. , Rahman D. , and Sanee A. , Knowledge and Skills as Main Predictors in Nurse Disaster Preparedness, JMMR (Jurnal Medicoeticolegal Dan Manajemen Rumah Sakit). (2022) 11, no. 3, 221–230, 10.18196/jmmr.v11i3.16292.

[40] Choi B. W. and Ha Y. , Factors Influencing on Core Competencies in Disaster Nursing by University Hospital Nurses’ Experiences of Caring for Patients With COVID-19, Journal of Korean Academy of Community Health Nursing. (2022) 33, 10.12799/jkachn.2022.33.2.247.

[41] Park H.-Y. and Kim J.-S. , Factors Influencing Disaster Nursing Core Competencies of Emergency Nurses, Applied Nursing Research. (2017) 37, 1–5, 10.1016/j.apnr.2017.06.004. PMC7135655 28985913

[42] Zhu D. , Li Y. , Chen Q. et al., Status and Influencing Factors of Clinical Nurses’ Disaster Nursing Competency: A Multicenter Cross-Sectional Study, BMC Nursing. (2025) 24, no. 1, 10.1186/s12912-025-03026-8. PMC11971766 40188344

[43] Jieun L. and Eunjoo L. , The Effects of Disaster Training Education on the Attitudes, Preparedness, and Competencies in Disaster Nursing of Hospital Nurses, Journal of Korean Academy of Community Health Nursing. (2020) 31, 491–502, 10.12799/JKACHN.2020.31.4.491.

[44] Mohamed N. A. , Abdel-Aziz H. R. , and Elsehrawy M. G. , Nursing Students’ Knowledge, Attitude, and Practice Regarding Disaster Preparedness: A Cross-Sectional Study, Risk Management and Healthcare Policy. (2023) 16, 2427–2437, 10.2147/RMHP.S435131. PMC10656855 38024484

